# Angiographic Lesion Morphology Provides Incremental Value to Generalize Quantitative Flow Ratio for Predicting Myocardial Ischemia

**DOI:** 10.3389/fcvm.2022.872498

**Published:** 2022-06-06

**Authors:** Jie Zhang, Na Zhao, Bo Xu, Lihua Xie, Weihua Yin, Yunqiang An, Hankun Yan, Yitong Yu, Bin Lu

**Affiliations:** ^1^Department of Radiology, Fuwai Hospital, Chinese Academy of Medical Sciences and Peking Union Medical College, Beijing, China; ^2^Department of Cardiology, Fuwai Hospital, Chinese Academy of Medical Sciences and Peking Union Medical College, Beijing, China

**Keywords:** lesion morphology, myocardial ischemia, quantitative flow ratio, fractional flow reserve, invasive coronary angiography

## Abstract

**Aim:**

The quantitative flow ratio (QFR) is favorable for functional assessment of coronary artery stenosis without pressure wires and induction of hyperemia. The aim of this study was to explore whether angiographic lesion morphology provides incremental value to generalize QFR for predicting myocardial ischemia in unselected patients.

**Methods:**

This study was a substudy to the CT-FFR CHINA trial, referring 345 participants from five centers with suspected coronary artery disease on coronary CT angiography for diagnostic invasive coronary angiography (ICA). Fractional flow reserve (FFR) was measured in all vessels with 30–90% diameter stenosis. QFR was calculated in 186 lesions from 159 participants in a blinded manner. In addition, parameters to characterize lesion features were recorded or measured, including left anterior descending arteries (LADs)-involved lesions, side branch located at stenotic lesion (BL), multiple lesions (ML), minimal lumen diameter (MLD), reference lumen diameter (RLD), percent diameter stenosis (%DS), lesion length (LL), and LL/MLD^4^. Logistic regression was used to construct two kinds of models by combining single or two lesion parameters with the QFR. The performances of these models were compared with that of QFR on a per-vessel level.

**Results:**

A total of 148 participants (mean age: 59.5 years; 101 men) with 175 coronary arteries were included for final analysis. In total, 81 (46%) vessels were considered hemodynamically significant. QFR correctly classified 82.29% of the vessels using FFR with a cutoff of 0.80 as reference standard. The area under the receiver operating characteristic curve (AUC) of QFR was 0.86 with a sensitivity, specificity, positive predictive value, and negative predictive value of 80.25, 84.04, 81.25, and 83.16%, respectively. The combined models (QFR + LAD + MLD, QFR + LAD + %DS, QFR + BL + MLD, and QFR + BL + %DS) outperformed QFR with higher AUCs (0.91 vs. 0.86, *P* = 0.02; 0.91 vs. 0.86, *P* = 0.02; 0.91 vs. 0.86, *P* = 0.02; 0.90 vs. 0.86, *P* = 0.03, respectively). Compared with QFR, the sensitivity of the combined models (QFR + BL and QFR + MLD) was improved (91.36 vs. 80.25%, 91.36 vs. 80.25%, respectively, both *P* < 0.05) without compromised specificity or accuracy.

**Conclusion:**

Combined with angiographic lesion parameters, QFR can be optimized for predicting myocardial ischemia in unselected patients.

## Introduction

Under the general trend of precise diagnosis and treatment, the stenosis-driven percutaneous coronary intervention has gradually evolved into ischemia-driven percutaneous coronary intervention. Assessment of functional significance is recommended for patients with intermediate coronary artery lesions (1, 2). Fractional flow reserve (FFR), which is invasively assessed during coronary angiography by advancing a wire with a pressure transducer toward the stenotic lesion, is the reference standard for lesion-specific ischemia evaluation (3–6). Many studies have shown that by implementing a strategy of FFR-guided percutaneous coronary intervention, the number of implanted stents is reduced, and clinical outcomes are significantly improved (7–10). However, there are many challenges with FFR measurement, such as wire cost, limitations associated with induction of hyperemia, and additional procedure time (11).

Recently, several image-based FFR methodologies have been proposed in an attempt to replace invasive FFR assessment (7, 12–20). These methodologies are mainly derived from different coronary examinations, including cardiac CT angiography (7, 12–18), invasive coronary angiography (ICA) (19–38), intravascular ultrasound (39), and intravascular optical coherence tomography (40–44). Among them, FFR derived from ICA has demonstrated feasibility for identifying ischemic coronary lesions with excellent diagnostic performance. There are two different principles for the calculation. One is based on simplistic fluid equations (20–34), and the other is based on computational fluid dynamics (19, 35–38). The latter principle depends on the whole anatomical geometry of the coronary vessel, while the former one depends on the previous datasets and the stenosis geometry. Quantitative flow ratio (QFR), which is based on simplistic fluid equations, has shown the potential for FFR alternatives in routine use with high efficiency. Correct 3D target vessel reconstruction from ICA image runs is required for QFR calculation (19, 34). To obtain the exact topology of the interrogated vessel, two angiographic projections at different angles ≥25° are selected. Due to the requirement of two optimal projections for the reconstruction of the entire target vessel, vessels with ostial stenosis, severe overlap, or tortuosity are usually not analyzable by QFR (33). Additionally, since only the main vessel of interest is reconstructed without any branches for QFR computation, stenosis involving both sides of a major shift (>1 mm) in reference diameter is not appropriate for QFR assessment (33, 34). A previous study has reported that approximately 15–20% of patients are excluded due to these strict exclusion criteria (45). When taking these ineligible cases into consideration, the diagnostic performance of QFR would be impaired. The WIFI II study has shown that for unselected patients, the sensitivity and area under the receiver operator characteristic curve (AUC) of QFR are 77% and 0.86, respectively (30), which is slightly compromised compared with QFR clinical trials (31). As a potential candidate to replace invasive FFR assessment, QFR should have excellent performance in unselected patients. Therefore, it is of great significance to generalize QFR for predicting myocardial ischemia.

It has been hypothesized that angiographic lesion morphology is helpful for QFR assessment because some information, such as side branches and lesion-involved vessels, is not taken into account for QFR computation. Therefore, the objective of this study was to evaluate the value of angiographic lesion morphology for QFR by constructing combined models for predicting myocardial ischemia in unselected patients.

## Materials and Methods

### Study Design and Study Population

**FIGURE 1:**
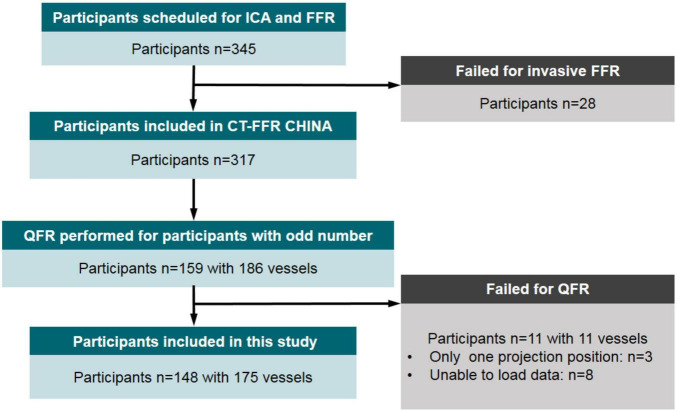
Flow diagram of participant inclusion. ICA, invasive (conventional) coronary angiography; FFR, fractional flow reserve; QFR, quantitative flow ratio.

This study was a substudy of the prospective and multicentered CT-FFR CHINA trial which is registered at www.ClinicalTrials.gov (NCT03692936). Participants underwent cardiac CT angiography ≤7 days before scheduled, non-emergent, and clinically indicated ICA between November 2018 and March 2020. Ref. (46) has explained the inclusion and exclusion criteria of the trial in detail. Inclusion criteria included participants aged ≥18 years and one or more lesions with 30–90% diameter stenosis in a ≥2.0 mm diameter vessel according to cardiac CT angiography, scheduled for ICA based on clinical evaluation. Clinical exclusion criteria included previous percutaneous coronary intervention or bypass surgery, previous myocardial infarction, acute myocardial infarction, allergy to contrast agent, and contraindications to beta-blockers, nitroglycerin, or adenosine, serum creatinine >150 μmol/L, or a glomerular filtration rate <45 ml/kg/1.73 m^2^, severe heart failure, and pregnant state. Cardiac CT angiography exclusion criteria were significant arrhythmia (atrial fibrillation) and poor image quality. Angiographic exclusion criteria were the failure of invasive FFR procedure and incomplete data for QFR computation. The flowchart related to this study is shown in Figure 1. With 28 patients in whom invasive FFR failed to be measured, 317 participants successfully underwent both ICA and FFR. In this study, half of the participants were scheduled for the QFR procedure to explore the value of angiographic lesion morphology for QFR. Therefore, these participants from five clinics were numbered in order, and participants with odd numbers were selected to calculate QFR and record lesion parameters. A total of 148 participants with 175 vessels were included for final analysis in this study. The diagnostic performances of the predictive models combining QFR with angiographic lesion parameters were assessed and compared with QFR. The study protocol was approved at the five centers by each of the local Institutional Review Boards, and participants provided written informed consent.

The ICA and FFR data were acquired and analyzed by a core laboratory in a blinded manner. QFR was computed offline by experienced technicians (L.X. and Z.S., both with more than 3 years of experience) from the cardiovascular intervention department who were blinded to FFR readings. The clinical data of the included participants were collected from electronic medical databases, including age, sex, body mass index, and high-risk factors for coronary artery disease.

### Invasive Coronary Angiography and Fractional Flow Reserve Measurement

Selective ICA was performed using standard catheterization according to the American College of Cardiology Guidelines for Coronary Angiography (47). At least two projection angles were acquired for the optimal view of the stenotic lesion. With clinical indication for FFR measurement, a pressure-monitoring guidewire (St. Jude Medical, Minneapolis, MN, United States) was advanced 1–2 cm distal to the lesion after administration of nitroglycerin. To achieve hyperemia, intravenous adenosine (160 μg/kg/min) was implemented. Pressure data were recorded for at least 3 s of stable value before adenosine administration and at least 10 s of stable value during hyperemia. FFR was defined as the ratio of mean distal coronary pressure to mean aortic pressure during maximum hyperemia. FFR ≤0.80 was considered hemodynamically significant (48).

### Quantitative Coronary Angiography Analysis

Quantitative coronary angiography (QCA) analysis was performed based on angiograms using commercial software (QAngioXA 7.3; Medis Medical Imaging System, Leiden, Netherlands). Observers only received diagnostic angiographic runs and were blinded to any potential treatment, FFR results, and QFR results. Optimal projections were selected for the most stenotic lesion, and indices including minimal lumen diameter (MLD), reference lumen diameter (RLD), and lesion length (LL) were measured for analysis. In addition, percent diameter stenosis (%DS) and lesion length/minimal luminal diameter^4^ (LL/MLD^4^) were derived from the above-related indices. Notably, proximal and distal measurement points were approximately 10 mm away from the start and end of the lesion, respectively, if possible. LAD-involved lesions were recorded because the severity of non-LAD involved lesions tended to be overestimated by anatomical parameters of QCA lesions compared with LAD-involved lesions. Lesions with side branch (BL) and multiple lesions with %DS >30% (ML) in one interrogated vessel were recorded as well.

### Quantitative Flow Ratio Computation

**FIGURE 2:**
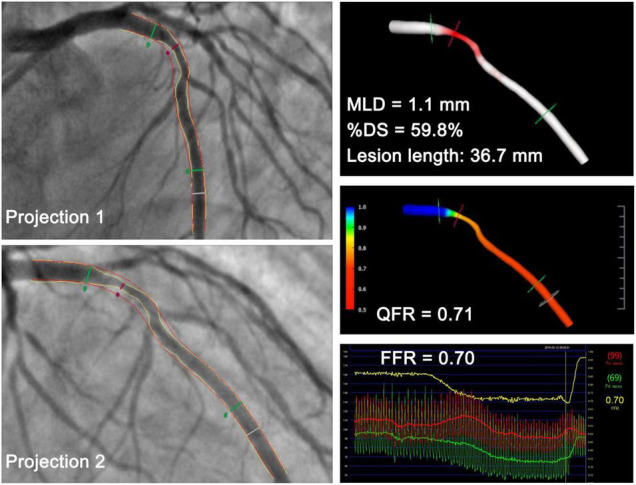
Representative case in a 53-year-old man with a minimal lumen diameter of 1.1 mm at the lesion site. QFR derived from the two projections was 0.71, which was in accordance with the FFR measurement of 0.70. MLD, minimal lumen diameter; %DS, percent diameter stenosis; QFR, quantitative flow ratio; FFR, fractional flow reserve.

The QFR was calculated offline through a commercial software package (AngioPlus, Pulse Medical Imaging Technology, Shanghai Co., Ltd., Shanghai, China) by experienced technicians who were certified for the software operation and blinded to the FFR readings. Before QFR computation, technicians were informed about the location where the operators had measured the FFR to allow comparison of the QFR to the FFR at the same vessel site. The vessel was reconstructed from two diagnostic angiographic projections ≥25° apart (Figure 2) without a side branch. The lumen contour was automatically delineated, and a manual correction was allowed in cases of poor angiographic image quality or vessel overlap. The reconstructed vessel was automatically divided into several subsegments along the arterial centerline, and the estimated contrast flow velocity was derived *via* a frame count method (19). The estimated contrast flow velocity was automatically converted into a virtual hyperemic flow velocity using a quadratic function. The pressure drop for each subsegment was calculated using the stenosis geometry and virtual hyperemic flow velocity (19). The pressure drop at every position with respect to the most proximal position was calculated by integrating the pressure drop of all subsegments proximal to that interrogated location (19). Finally, the pressure drop along the segmented vessel enabled QFR reading along the vessel. QFR ≤0.80 was used as the diagnostic cutoff value.

### Statistical Analysis

SPSS 22.0 (IBM SPSS Statistics) and MedCalc 18.2 (MedCalc Software) software programs were applied for statistical analysis. All analyses were performed on a per-vessel level. The normality of quantitative data was assessed using the Kolmogorov–Smirnov test. As for quantitative variables with normal distribution, data were expressed as mean ± standard deviation and compared using *t*-tests. Otherwise, data were expressed as median with interquartile ranges and compared using the Kruskal–Wallis H test. In regard to categorical variables, the Chi-square test or Fisher exact test was used to compare rates as appropriate. The correlation between QFR and FFR was assessed with the Spearman correlation coefficient. A Bland–Altman plot and Wilcoxon signed-rank test were used to visualize and compare QFR and FFR.

All interrogated vessels were assigned to either the ischemia or non-ischemia group based on FFR. Two kinds of logistic regression models with two variables or three variables (one variable was QFR) were constructed to explore the incremental value of angiographic lesion morphology for QFR. Variables that were statistically significant in bivariate logistic regression analyses were included in trivariate logistic regression analyses. The diagnostic accuracy, sensitivity, specificity, positive predictive value (PPV), and negative predictive value (NPV) of these models as well as QFR were calculated and analyzed. The AUC values for the combined models and QFR were compared with the methods of DeLong et al. (49), which were implemented through MedCalc 18.2 software. Statistical significance was assumed at *P* < 0.05.

## Results

### Participant Characteristics

A total of 11 participants with 11 vessels failed for QFR computation mainly due to a lack of proper position or calibrated data. Therefore, 148 participants with 175 vessels were included in this study. The detailed demographics of the 148 participants (mean age of 59.5 ± 9.7 years with 101 males) are presented in Table 1. The interrogated vessels included 119 LAD arteries, 29 left circumflex arteries, and 27 right coronary arteries. Lesions in side branches were classified into the corresponding main vessels.

**TABLE 1:** Baseline clinical characteristics.

Parameter	148 patients
Age	59.5 ± 9.7
Male	101 (68.24)
BMI (kg/m^2^)	25.72 ± 3.09
**Risk factors**	
Hypertension	98 (66.22)
Diabetes mellitus	43 (29.05)
Dyslipidemia	99 (66.89)
Obesity	14 (14.15)
Current smokers	43 (29.05)
Family history of CAD	16 (16.15)
eGFR (ml/min/1.73 m^2^)	95.33 ± 26.41
**Symptom characteristics**	
Stable angina	96 (64.86)
Unstable angina	32 (21.62)
Other symptoms	20 (13.51)

*BMI, body mass index; CAD, coronary artery diseases; eGFR, estimated glomerular filtration rate.*

*Data are means ± standard deviations (SD) or n (%).*

*Other symptoms indicate chest distress, fatigue, suffocation, and so on.*

### Fractional Flow Reserve Characteristics and Angiographic Findings

**FIGURE 3:**
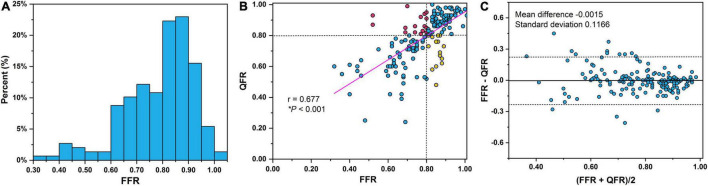
**(A)** Distribution of FFR measurements. Notably, 12.6% (*n* = 22) of the lesions were in the FFR 0.75–0.80 interval. Correlations **(B)** and agreement **(C)** between QFR and FFR. Dashed lines in Bland–Altman plot illustrate the mean difference ± 1.96 standard deviation. QFR, quantitative flow ratio; FFR, fractional flow reserve.

The lesions had a mean FFR of 0.78 ± 0.13 and a median FFR of 0.81 (interquartile range: 0.70–0.88) (Figure 3A). A positive FFR (≤0.80) was identified in 46.29% (*n* = 81) of the participants. The comparisons of lesion morphology described by lesion location, lesion features, and QCA indices between ischemic and non-ischemic lesions are displayed in Table 2. There were significantly fewer ischemic lesions in left circumflex arteries and right coronary arteries than in LAD arteries. Moreover, 57.14% of vessels (*n* = 100) had side branches at the lesion site and 36% of vessels (*n* = 63) had multiple lesions with %DS >30%. BL and ML were more common in the ischemic group (both *P* < 0.0001). The mean MLD, RLD, %DS, and LL were 1.37 ± 0.46 mm, 2.66 ± 0.60 mm, 48.29 ± 13.2, and 10.52 ± 4.55 mm, respectively. Overall, lesions with a smaller MLD (1.11 vs. 1.61 mm, *P* < 0.0001), smaller RLD (2.50 vs. 2.79 mm, *P* = 0.002), and longer LL (11.64 vs. 9.46 mm, *P* < 0.0001) were more prone to causing myocardial ischemia. Correspondingly, the %DS (55.52 vs. 41.72, *P* < 0.0001) and the LL/MLD^4^ Poiseuille-based coronary angiographic index (7.67 vs. 1.47, *P* < 0.0001) were greater in the myocardial ischemia group compared with the non-ischemia group.

**TABLE 2:** Angiographic and FFR findings (*n* = 175).

Parameter	All arteries	FFR ≤ 0.8	FFR > 0.8	*P*-value
No. of vessels	175	81 (46.29)	94 (53.71)	0.36
FFR index	0.81 (0.70–0.88)	0.67 ± 0.11	0.88 ± 0.05	<0.0001
QFR index	0.82 (0.70–0.91)	0.69 ± 0.15	0.87 ± 0.10	<0.0001
**Lesion location**				
LAD	119 (68)	69 (57.98)	50 (40.02)	0.10
LCX	29 (16.57)	8 (27.59)	21 (72.41)	0.03
RCA	27 (15.43)	4 (14.81)	23 (85.19)	0.001
**Lesion features**				
BL	100 (57.14)	61 (61)	39 (39)	<0.0001
ML	63 (36)	43 (68.25)	20 (31.75)	<0.0001
**QCA indices**				
MLD (mm)	1.37 ± 0.46	1.11 ± 0.37	1.61 ± 0.40	<0.0001
RLD (mm)	2.66 ± 0.60	2.50 ± 0.66	2.79 ± 0.52	0.002
% DS	48.29 ± 13.2	55.52 ± 10.65	41.72 ± 11.97	<0.0001
LL (mm)	10.52 ± 4.55	11.64 ± 4.81	9.46 ± 4.11	<0.0001
LL/MLD^4^	3.29 (1.10–10.17)	7.67 (3.45–16.97)	1.47 (0.67–3.28)	<0.0001

*FFR, fractional flow reserve; QFR, quantitative flow ratio; LAD, left anterior descending artery; LCX, left circumflex artery; RCA, right coronary artery; MLD, minimal lumen diameter; RLD, reference lumen diameter; %DS, percent diameter stenosis; LL, lesion length; BL, side branch at the lesion site; ML, multiple lesions with %DS >30%.*

*Data are mean ± SD or n (%) or median (interquartile range), and P-values were calculated with t-tests or Chi-square tests.*

### Performance of Quantitative Flow Ratio

The correlation and agreement between QFR and FFR are shown in Figures 3B,C, respectively. A good correlation (Figure 3B) and agreement (Figure 3C) of QFR and FFR were observed with a correlation coefficient of 0.677 and a mean difference of −0.0015. The mean QFR and median QFR were 0.78 ± 0.15 and 0.82 (interquartile range: 0.70–0.91), respectively. The best cut-off value of QFR was 0.795. QFR correctly classified 82.29% of the vessels using FFR with a cutoff of 0.80 as the reference standard. The AUC of QFR was 0.86 (95% confidence interval, 0.80–0.91) with a sensitivity, specificity, PPV, and NPV of 80.25, 84.04, 81.25, and 83.16%, respectively.

### Performance of the Predictive Models With a Single Lesion Parameter Added to Quantitative Flow Ratio

**FIGURE 4:**
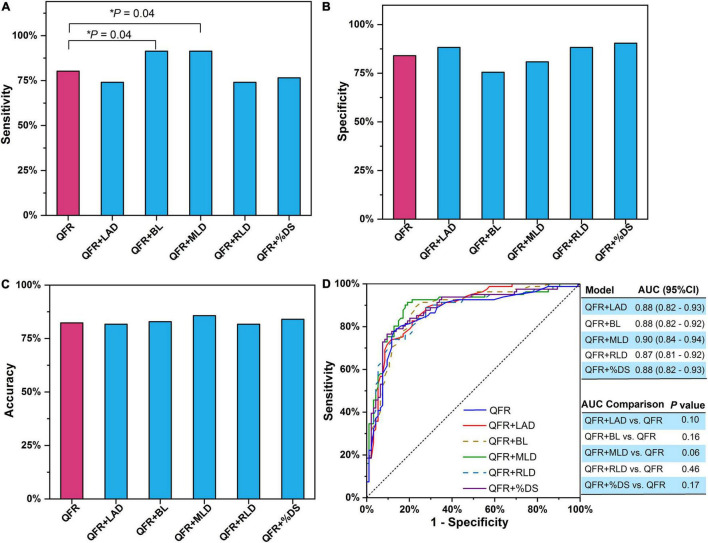
Single lesion parameter combined with QFR for predicting myocardial ischemia. **(A)** Sensitivity. **(B)** Specificity. **(C)** Accuracy. **(D)** AUC. The *P*-value reflects the AUC comparison between the combined model and QFR. QFR, quantitative flow ratio; LAD, left anterior descending artery; MLD, minimal lumen diameter; RLD, reference lumen diameter; %DS, percent diameter stenosis; BL, side branch at lesion site; ML, multiple lesions with %DS >30%; AUC, area under receiver operator characteristic curve; CI, confidence interval. The symbol * means the related P value is statistically significant with less than 0.05.

Bivariate logistic regression was applied to construct predictive models combining a single lesion parameter with QFR for the prediction of myocardial ischemia (Table 3). Among the eight lesion parameters used in this study (i.e., LAD, BL, ML, MLD, RLD, %DS, LL, and LL/MLD^4^ with the former three being dichotomous variables), LAD (odds ratio = 0.233, *P* = 0.002), BL (odds ratio = 0.330, *P* = 0.006), MLD (odds ratio = 0.090, *P* < 0.0001), RLD (odds ratio = 0.407, *P* = 0.009), and %DS (odds ratio = 1.070, *P* = 0.001) were effective predictors for predicting myocardial ischemia when added to QFR. The accuracy, sensitivity, specificity, PPV, and NPV of the five models with effective predictors are presented in Table 4. Compared with QFR, the sensitivity of the combined models (QFR + BL and QFR + MLD) was significantly improved without compromised specificity and accuracy, as shown in Figures 4A–C. Furthermore, the AUC values and accuracy of the two combined models were higher than those of QFR (AUC: 0.88 vs. 0.86 and 0.90 vs. 0.86, respectively; accuracy: 82.86% vs. 82.29% and 85.72% vs. 82.29%, respectively), but they were not statistically significant (all *P* > 0.05) (Figure 4D).

**TABLE 3:** Logistic regression model with a single lesion parameter added to QFR.

Predictive model	OR (95% CI)	*P*-value of lesion index
QFR + LAD	0.233 (0.093–0.587)	**0.002**
QFR + BL	0.330 (0.149–0.732)	**0.006**
QFR + ML	0.653 (0.287–1.486)	0.31
QFR + MLD	0.090 (0.028–0.293)	**<0.0001**
QFR + RLD	0.407 (0.208–0.795)	**0.009**
QFR + %DS	1.070 (1.028–1.114)	**0.001**
QFR + LL	1.058 (0.963–1.162)	0.24
QFR + LL/MLD^4^	1.006 (0.994–1.018)	0.31

*QFR, quantitative flow ratio; LAD, lesion in left anterior descending artery; BL, side branch at the lesion site; ML, multiple lesions with %DS >30%; MLD, minimal lumen diameter; RLD, reference lumen diameter; %DS, percent diameter stenosis; LL, lesion length; OR, odds ratio; CI, confidence interval. The bold type means the related P value is statistically significant with less than 0.05.*

**TABLE 4:** Performance of combined models with single lesion parameter added to QFR.

Parameter	AUC	Accuracy (%)	Sensitivity (%)	Specificity (%)	PPV (%)	NPV (%)
QFR	0.86	82.29	80.25	84.04	81.25	83.16
QFR + LAD	0.88	81.71	74.07	88.30	84.51	79.81
QFR + BL	0.88	82.86	91.36	75.53	76.29	91.03
QFR + MLD	0.90	85.72	91.36	80.85	80.43	92.57
QFR + RLD	0.87	81.71	74.07	88.30	84.51	79.81
QFR + %DS	0.88	84.00	76.54	90.04	87.32	81.73

*QFR, quantitative flow ratio; LAD, lesion in left anterior descending artery; BL, side branch at the lesion site; MLD, minimal lumen diameter; RLD, reference lumen diameter; %DS, percent diameter stenosis; AUC, area under the receiver operating characteristic curve; PPV, positive predictive value; NPV, negative predictive value.*

### Performance of the Predictive Model With Two Lesion Parameters Added to Quantitative Flow Ratio

**FIGURE 5:**
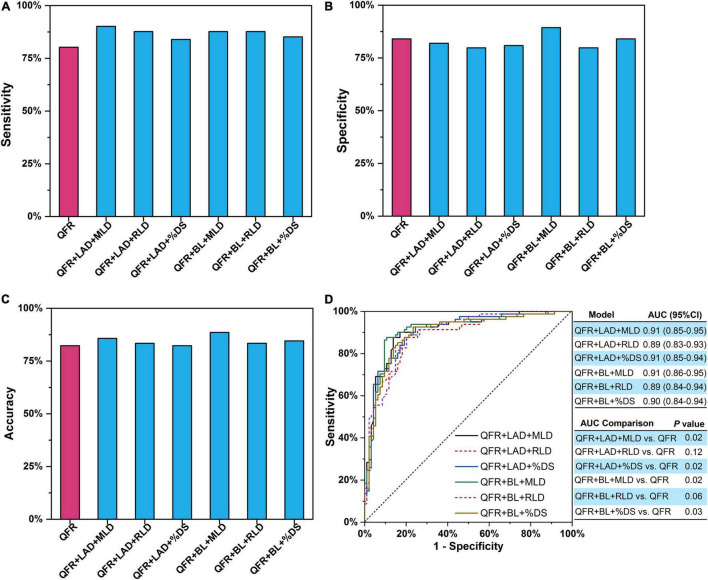
Two lesion parameters combined with QFR for predicting myocardial ischemia. **(A)** Sensitivity. **(B)** Specificity. **(C)** Accuracy. **(D)** AUCs. The *P*-value reflects the AUC comparison between the combined model and QFR. QFR, quantitative flow ratio; LAD, left anterior descending artery; MLD, minimal lumen diameter; RLD, reference lumen diameter; %DS, percent diameter stenosis; BL, side branch at lesion site; ML, multiple lesions with %DS >30%; AUC, area under receiver operator characteristic curve; CI, confidence interval.

Trivariate logistic regression was applied to construct predictive models combining two lesion parameters with QFR for the prediction of myocardial ischemia (Table 5). Five lesion parameters (i.e., LAD, BL, MLD, RLD, and %DS) were selected for trivariate logistic regression analyses because they were effective predictors in bivariate logistic regression. Considering the internal relation among MLD, RLD, and %DS, the three parameters were not combined in one model. Therefore, six combined predictive models were constructed with two lesion parameters as predictors added to QFR. The accuracy, sensitivity, specificity, PPV, and NPV of the six combined models are presented in Table 6. In contrast to QFR, the AUCs of the QFR + LAD + MLD, QFR + LAD + %DS, QFR + BL + MLD, and QFR + BL + %DS combined models were all significantly improved (0.91 vs. 0.86, *P* = 0.02; 0.91 vs. 0.86, *P* = 0.02; 0.90 vs. 0.86, *P* = 0.03, respectively) without compromising other performances (Figure 5D). Notably, the accuracy, sensitivity, specificity, PPV, and NPV of the QFR + BL + MLD combined model were higher than those of QFR (Table 6 and Figure 5), but they were not statistically significant (both *P* > 0.05).

**TABLE 5:** Logistic regression model with two lesion parameters added to QFR.

	Lesion parameters	OR (95% CI)	*P*-value of lesion parameter
QFR+	LAD	0.233 (0.085–0.644)	**0.005**
	MLD	0.095 (0.029–0.317)	**<0.0001**
QFR+	LAD	0.280 (0.108–0.724)	**0.009**
	RLD	0.493 (0.246–0.985)	**0.045**
QFR+	LAD	0.165 (0.060–0.452)	**<0.0001**
	%DS	1.082 (1.037–1.129)	**<0.0001**
QFR+	BL	0.245 (0.100–0.602)	**0.002**
	MLD	0.074 (0.022–0.245)	**<0.0001**
QFR+	BL	0.270 (0.115–0.630)	**0.002**
	RLD	0.344 (0.169–0.700)	**0.003**
QFR+	BL	0.287 (0.123–0.668)	**0.004**
	%DS	1.075 (1.031–1.120)	**0.001**

*QFR, quantitative flow ratio; LAD, lesion in left anterior descending artery; BL, side branch at the lesion site; MLD, minimal lumen diameter; RLD, reference lumen diameter; %DS, percent diameter stenosis; OR, odds ratio; CI, confidence interval.*

*The bold type means the related P value is statistically significant with less than 0.05.*

**TABLE 6:** Performance of combined models with two lesion parameters added to QFR.

Parameter	AUC	Accuracy (%)	Sensitivity (%)	Specificity (%)	PPV (%)	NPV (%)
QFR	0.86	82.29	80.25	84.04	81.25	83.16
QFR + LAD + MLD	0.91	85.71	90.12	81.91	81.11	90.59
QFR + LAD + RLD	0.89	83.43	87.65	79.79	78.89	88.24
QFR + LAD + %DS	0.91	82.29	83.95	80.85	79.07	85.39
QFR + BL + MLD	0.91	88.57	87.65	89.36	87.65	89.36
QFR + BL + RLD	0.89	83.43	87.65	79.79	78.89	88.24
QFR + BL + %DS	0.90	84.57	85.19	84.04	82.14	86.81

*QFR, quantitative flow ratio; LAD, lesion in left anterior descending artery; MLD, minimal lumen diameter; RLD, reference diameter; %DS, diameter stenosis; BL, side branch at the lesion site; AUC, area under the receiver operating characteristic curve; PPV, positive predictive value; NPV, negative predictive value.*

## Discussion

Prior studies have demonstrated that QFR is favorable for the assessment of coronary artery stenosis-caused myocardial ischemia. However, there are strict conditions for QFR application. In general, patients with severe vessel overlap or tortuosity at the stenosed segments are excluded from QFR computation. Similarly, main vessels with stenosed side branches downstream of the interrogated lesion are also not appropriate for QFR analysis. Under these predefined strict exclusion criteria (33), the diagnostic performance of QFR is excellent with an AUC value up to 0.92, which has been demonstrated in previous clinical trials (31, 33). The performance of QFR is slightly compromised with sensitivity and AUC of 77% and 0.86, respectively, in unselected consecutive participants (30). In this study, a common analysis protocol was applied to all lesion subsets without excluding specific lesion types or localizations. Similar to the WIFI II study (30), the AUC of QFR was 0.86 in this study due to inconsistent exclusion criteria. When constructing predictive models with two lesion parameters added to QFR (QFR + LAD + MLD, QFR + LAD + %DS, or QFR + BL + MLD), the diagnostic performance was significantly improved with AUC up to 0.91. In addition, with three lesion parameters added to QFR (QFR + LAD + BL + MLD), an AUC of 0.92 (95% CI: 0.87–0.95) was achieved (Table 7). Furthermore, the sensitivity of the models (QFR + BL and QFR + MLD) was significantly higher than that of QFR (91.36 vs. 80.25%, *P* = 0.04 and 91.36 vs. 80.25%, *P* = 0.04, respectively) without compromising AUC, accuracy, and specificity.

**TABLE 7:** Logistic regression model with three lesion parameters added to QFR.

	Lesion parameter	OR (95% CI)	*P*-value of lesion parameter
QFR+	LAD	3.033 (1.030–8.927)	**0.04**
	BL	0.324 (0.126–0.834)	**0.02**
	MLD	0.081 (0.024–0.273)	**<0.0001**
QFR+	LAD	2.466 (0.878–6.929)	0.09
	BL	0.351 (0.143–0.861)	**0.02**
	RLD	0.411 (0.197–0.859)	**0.02**
QFR+	LAD	4.767 (1.646–13.811)	**0.004**
	BL	0.414 (0.168–1.020)	0.06
	%DS	1.038 (1.038–1.130)	**<0.0001**

*QFR, quantitative flow ratio; LAD, lesion in left anterior descending artery; BL, side branch at the lesion site; MLD, minimal lumen diameter; RLD, reference lumen diameter; %DS, percent diameter stenosis; OR, odds ratio; CI, confidence interval.*

*The bold type means the related P value is statistically significant with less than 0.05.*

There was no subjective factor affecting the LAD and BL lesion parameters because they were obtained only by recording whether it was a LAD-involved lesion and whether a BL existed at the lesion site. The MLD and %DS parameters were measured using widespread QCA software, which was installed in every cardiac catheterization laboratory. Thus, there were few interference factors when obtaining these predictive parameters for constructing the combined models. It was feasible for the clinical staff to adopt the models without any change in the clinical pathway. Furthermore, there were 22 participants with FFR between 0.75 and 0.80, and 9 false positives were assessed by QFR. Notably, only 5 false positives were assessed using the QFR + LAD + MLD model, which showed the potential to improve the diagnostic performance of QFR within gray FFR. Overall, this study demonstrates that angiographic lesion morphology provided incremental value to generalize QFR for predicting myocardial ischemia in unselected patients. The setup of this study generalizes applicable conditions for QFR in normal clinical practice.

## Limitations

There were several limitations in this study. As participants were referred to ICA based on coronary CT angiography with 30–90% diameter stenosis in a ≥2.0 mm diameter vessel, some vessels, which did not have a %DS of >30% on ICA, were also eligible for this study procedure. In addition, in-procedure QFR was not feasible in this study, which may compromise the diagnostic performance of QFR because direct feedback from a percutaneous coronary intervention operator after early identification of insufficient angiographic quality may improve the overall performance of QFR. As the sample size of vessels with LAD-involved lesions was larger than that of left circumflex arteries and right coronary arteries, a larger study population that includes more lesions in left circumflex arteries or right coronary arteries is needed to confirm the above results. Compared with LAD-involved lesions, anatomical parameters of QCA lesions tended to overestimate the severity of non-LAD lesions, which indicates that the best cutoff value of QFR or predictive models for LAD may differ from that for non-LAD. Furthermore, patients with different symptom characteristics were analyzed as a whole when constructing models. Stratification analysis according to stable angina vs. unstable angina was implemented in Supplement Appendix. The results indicate that for patients with unstable angina, QFR correctly classified 32 of the 36 vessels with 16 true positives, 16 true negatives, 1 false negative, and 3 false positives. Due to the lack of enough samples, there is less point to construct the combined models on patients with unstable angina in this study. Besides, none of the selected parameters were effective predictors for the combined models. As for the patients with stable angina, the combined model QFR + BL + MLD showed the best performance compared with QFR, which was similar to the analysis without stratification. Further study should demonstrate whether it is required to refine these models according to different lesion locations and symptom characteristics. When calculating QFR, the side branch was not included in vessel reconstruction, which may have decreased the diagnostic accuracy in the assessment of bifurcation lesions. Therefore, predictive models should be constructed, especially for bifurcation lesions, in future studies.

## Conclusion

The predictive model combining lesion parameters with QFR improves the diagnostic performance of QFR. Angiographic lesion morphology provides incremental value to generalize QFR for predicting myocardial ischemia.

## Data Availability Statement

The original contributions presented in this study are included in the article/Supplementary Material, further inquiries can be directed to the corresponding author.

## Ethics Statement

The studies involving human participants were reviewed and approved by the Fuwai Hospital, Beijing, China; Chaoyang Hospital, Beijing, China; Qi Lu Hospital, Shan Dong University, Jinan, Shandong, China; Sir Run Run Shaw Hospital, Zhe Jiang University School of Medicine, Hangzhou, Zhejiang, China; Teda International Cardiovascular Hospital, Tianjin, China. The patients/participants provided their written informed consent to participate in this study. Written informed consent was obtained from the individual(s) for the publication of any potentially identifiable images or data included in this article.

## Author Contributions

BL and JZ conceived the study design. BX and LX conducted QFR measurements. BL, JZ, NZ, WY, HY, and YY analyzed the data. JZ, YA, and NZ performed the statistical analysis. BL, JZ, and NZ interpreted the results. JZ drafted the manuscript. BL, JZ, WY, and NZ edited and revised the manuscript. All authors read and approved the final manuscript.

## Conflict of Interest

The authors declare that the research was conducted in the absence of any commercial or financial relationships that could be construed as a potential conflict of interest.

## Publisher’s Note

All claims expressed in this article are solely those of the authors and do not necessarily represent those of their affiliated organizations, or those of the publisher, the editors and the reviewers. Any product that may be evaluated in this article, or claim that may be made by its manufacturer, is not guaranteed or endorsed by the publisher.

## Abbreviations

%DS: percent diameter stenosis
AUC: area under the receiver operator characteristic curve
BL: side branch at lesion site
FFR: fractional flow reserve
ICA: invasive coronary angiography
LAD: left anterior descending artery
LL: lesion length
ML: multiple lesions with %DS > 30%
MLD: minimal lumen diameter
QCA: quantitative coronary angiography
QFR: quantitative flow ratio
RLD: reference lumen diameter.
