# Supplementary material for: Angiographic Lesion Morphology Provides Incremental Value to Generalize Quantitative Flow Ratio for Predicting Myocardial Ischemia
Source: Front Cardiovasc Med. 2022 Jun 6;9:872498. doi: 10.3389/fcvm.2022.872498 (PMC9207314; doi:10.3389/fcvm.2022.872498)
Supplement: Supplementary file 1 [file Table_1.docx]

SUPPLEMENT

Angiographic lesion morphology provides incremental value to generalize QFR for predicting myocardial ischemia

Jie Zhang^1^, Na Zhao^1^, Bo Xu^2^, Lihua Xie^2^, Weihua Yin^1^, Yunqiang An^1^, Hankun Yan^1^, Yitong Yu^1^ and Bin Lu^1*^

^1^Department of Radiology, Fuwai Hospital, Chinese Academy of Medical Sciences and Peking Union Medical College, Beijing, China

^2^Department of Cardiology, Fuwai Hospital, Chinese Academy of Medical Sciences and Peking Union Medical College, Beijing, China

# SI Appendix I: Baseline clinical characteristics

There were 96 patients (64.86%) with stable angina and 32 patients (21.62%) with unstable angina in this study. The age for patients with stable angina were higher than that for patients with unstable angina (60.5 ± 9.7 vs. 55.6 ± 9.6, P = 0.02). There were no other significant differences between the two groups, as shown in Table 1.

| **Table 1\|** Baseline clinical characteristics | | | |
| --- | --- | --- | --- |
| **Parameter** | **Stable angina** | **Unstable angina** | **P value** |
| No. of patients | 96 (64.86) | 32 (21.62) | **<0.0001** |
| Age | 60.5 ± 9.7 | 55.6 ± 9.6 | **0.02** |
| Gender(Male) | 65 (67.71) | 20 (62.50) | 0.59 |
| BMI | 25.9 ± 3.2 | 25.9 ± 2.6 | 0.89 |
| Risk factors |  |  |  |
| Hypertension | 60 (62.50) | 24 (75.00) | 0.18 |
| Diabetes mellitus | 27 (28.13) | 10 (31.25) | 0.74 |
| Dyslipidemia | 67 (70.53) | 22 (68.75) | 0.91 |
| Obesity | 25 (26.04) | 8 (25.00) | 0.91 |
| Current smokers | 11 (34.38) | 7 (21.88) | 0.24 |
| Family history of CAD | 31 (32.29) | 4 (12.50) | 0.88 |
| eGFR | 96.4 ± 25.9 | 91.8 ± 24.5 | 0.38 |
| *Note.—BMI, body mass index; CAD, coronary artery diseases; eGFR, estimated glomerular filtration rate.*  *Data are means ± standard deviations (SD) or n (%).*  *There were 20 (13.51%) patients with other symptoms such as chest distress, fatigue, suffocation.* | | | |

# SI Appendix II: Angiographic and FFR findings on patients with stable angina (n=116)

In regards to angiographic and FFR findings, only patients with stable angina were compared between the ischemic group and non-ischemic group because the number of patients with unstable angina (32 patients with 17 positive vessels and 19 negative vessels) was small. As shown in Table 2, ischemic lesions were significantly fewer in left circumﬂex arteries and right coronary arteries than that in LAD. 57.76% vessels (n = 67) had side branches at the lesion site and 38.79% vessels (n = 45) had multiple lesions with %DS > 30% (ML). ML was more common in the ischemic group ( P = 0.02). The mean MLD, RLD, %DS and LL were 1.39 ± 0.47 mm, 2.69 ± 0.60 mm, 48.28 ± 13.30 and 10.41 ± 4.68 mm, respectively. Overall, lesions with smaller MLD (1.08 vs. 1.63 mm, P < 0.0001), smaller RLD (2.51 vs. 2.83 mm, P = 0.004) and longer LL (11.60 vs. 9.49 mm, P < 0.02) were more prone to causing myocardial ischemia. Correspondingly, %DS (56.31 vs. 41.98, P < 0.0001) and the LL/MLD^4^ Poiseuille-based coronary angiographic index (7.70 vs. 1.34, P < 0.0001) were larger in myocardial ischemia group.

| **Table 2\|** Angiographic and FFR findings on patients with stable angina (n=116) | | | | |
| --- | --- | --- | --- | --- |
| **Parameter** | **All arteries** | **FFR <= 0.8** | **FFR > 0.8** | **P value** |
| No. of vessels | 116 | 51 (43.97) | 65 (56.03) | 0.19 |
| FFR index | 0.82(0.70-0.89) | 0.65 ± 0.13 | 0.88 ± 0.05 | <0.0001 |
| QFR index | 0.82(0.69-0.90) | 0.67 ± 0.17 | 0.86 ± 0.10 | <0.0001 |
| Lesion location |  |  |  |  |
| LAD | 76 (65.52) | 41 (53.95) | 35 (46.05) | 0.002 |
| LCX | 22 (18.97) | 7 (31.82) | 15 (68.18) | - |
| RCA | 18 (15.52) | 3 (33.33) | 15 (66.67) | - |
| Lesion features |  |  |  |  |
| BL | 67 (57.76) | 40 (59.70) | 27 (40.30) | 0.10 |
| ML | 45 (38.79) | 28 (62.22) | 17 (37.78) | 0.02 |
| QCA Indices |  |  |  |  |
| MLD (mm) | 1.39 ± 0.47 | 1.08 ± 0.38 | 1.63 ± 0.38 | <0.0001 |
| RLD (mm) | 2.69 ± 0.60 | 2.51 ± 0.68 | 2.83 ± 0.49 | 0.004 |
| %DS | 48.28 ± 13.30 | 56.31 ± 12.31 | 41.98 ± 10.38 | <0.0001 |
| LL (mm) | 10.41 ± 4.68 | 11.60 ± 5.31 | 9.49 ± 3.91 | 0.02 |
| LL/MLD^4^ | 2.99(1.11-9.09) | 7.70(3.33-18.44) | 1.34(0.60-2.93) | <0.0001 |
| *Note.—FFR, fractional flow reserve; QFR, quantitative flow ratio; LAD, left anterior descending artery; LCX, left circumflex artery; RCA, right coronary artery; MLD, minimal lumen diameter; RLD, reference lumen diameter; %DS, percent diameter stenosis; LL, lesion length; BL, side branch at lesion site; ML, multiple lesions with %DS > 30%.*  *Data are mean ± SD or n (%) or median (interquartile range), and P values were calculated with paired t tests or Chi-square tests.* | | | | |

# SI Appendix III: Logistic regression model with a single lesion parameter added to QFR

Bivariate logistic regression was applied to construct predictive models combining a single lesion parameter with QFR for the prediction of myocardial ischemia (Table 3). As for the patients with stable angina, among the eight lesion parameters used in the present study (i.e., LAD, BL, ML, MLD, RLD, %DS, LL and LL/MLD^4^ with the former three being dichotomous variables), LAD (odds ratio = 0.334, P = 0.04), BL (odds ratio = 0.261, P = 0.008), MLD (odds ratio = 0.055, P < 0.0001), RLD (odds ratio = 0.368, P = 0.02), %DS (odds ratio = 1.085, P = 0.001) and LL/MLD^4^ (odds ratio = 1.219, P = 0.002) were effective predictors for predicting myocardial ischemia when added to QFR. As for the patients with unstable angina, among the eight lesion parameters used in the present study (i.e., LAD, BL, ML, MLD, RLD, %DS, LL and LL/MLD^4^), none of them were able to predict myocardial ischemia as the P value of the lesion indices in each model were all larger than 0.05 (Supplement SI Appendix III, Table 3).

| **Table 3\|** Logistic regression model with a single lesion parameter added to QFR | | | | |
| --- | --- | --- | --- | --- |
| **Predictive model** | **Stable angina** | | **Unstable angina** | |
|  | **OR (95% CI)** | ***P* value of lesion index** | **OR (95% CI)** | ***P* value of lesion index** |
| QFR+LAD | 0.334 (0.118 - 0.941) | **0.04** | 0.236 (0.015 - 3.817) | 0.31 |
| QFR+BL | 0.261 (0.097 - 0.707) | **0.008** | 0.874 (0.108 - 7.068) | 0.90 |
| QFR+ML | 0.833 (0.313 - 2.221) | 0.72 | 0.134 (0.002 - 8.533) | 0.34 |
| QFR+MLD | 0.055 (0.012 - 0.262) | **<0.0001** | 0.508 (0.017 - 15.560) | 0.70 |
| QFR+RLD | 0.368 (0.164 - 0.824) | **0.02** | 0.323 (0.030 - 3.473) | 0.35 |
| QFR+%DS | 1.085 (1.032 - 1.141) | **0.001** | 1.003 (0.913 - 1.103) | 0.94 |
| QFR+LL | 1.071 (0.963 - 1.191) | 0.21 | 0.866 (0.649 - 1.154) | 0.33 |
| QFR+LL/MLD^4^ | 1.219 (1.074 - 1.384) | **0.002** | 0.972 (0.850 - 1.111) | 0.67 |
| *Note.—QFR, quantitative flow ratio; LAD, lesion in left anterior descending artery; BL, side branch at lesion site; ML, multiple lesions with %DS > 30%; MLD, minimal lumen diameter; RLD, reference lumen diameter; %DS, percent diameter stenosis; LL, lesion length; OR, odd ratio; CI, confidence interval.* | | | | |

# SI Appendix IV: Performance of combined models on patients with stable angina

The accuracy, sensitivity, specificity, PPV and NPV of the five models with effective predictors are presented in Table 4. Compared to QFR, the AUC of the combined model (QFR+MLD) was significantly improved without compromised accuracy, specificity and sensitivity (AUC: 0.90 vs. 0.85, P = 0.04; Accuracy: 85.34 vs. 81.90, P = 0.49; Sensitivity: 90.20 vs. 80.39, P = 0.16; Specificity: 81.54 vs. 83.08, P = 0.82).

| **Table 4\|** Performance of combined models on patients with stable angina | | | | | | |
| --- | --- | --- | --- | --- | --- | --- |
| **Predictive model** | **AUC** | **Accuracy**  **(%)** | **Sensitivity**  **(%)** | **Specificity**  **(%)** | **PPV**  **(%)** | **NPV**  **(%)** |
| QFR | 0.85 | 81.90 | 80.39 | 83.08 | 78.85 | 84.38 |
| QFR+LAD | 0.86 | 81.90 | 70.59 | 90.77 | 85.71 | 79.73 |
| QFR+BL | 0.87 | 81.90 | 78.43 | 84.62 | 80.00 | 83.33 |
| QFR+MLD | 0.90 | 85.34 | 90.20 | 81.54 | 79.31 | 91.38 |
| QFR+RLD | 0.87 | 81.03 | 90.20 | 73.85 | 73.02 | 90.57 |
| QFR+%DS | 0.88 | 86.21 | 84.31 | 87.69 | 84.31 | 87.69 |
| QFR+LL/MLD^4^ | 0.89 | 84.48 | 88.23 | 81.54 | 78.95 | 89.83 |
| *Note.—QFR, quantitative flow ratio; LAD, lesion in left anterior descending artery; BL, side branch at the lesion site; MLD, minimal lumen diameter; RLD, reference lumen diameter; %DS, percent diameter stenosis; AUC, area under the receiver operating characteristic curve; PPV, positive predictive value; NPV, negative predictive value.* | | | | | | |

# SI Appendix V: Logistic regression model on patients with stable angina

Trivariate logistic regression for patients with stable angina was applied to construct predictive models by combining two lesion parameters with QFR for the prediction of myocardial ischemia (Table 5). Six lesion parameters (i.e., LAD, BL, MLD, RLD, %DS and LL/MLD^4^) were selected for trivariate logistic regression analyses because they were effective predictors in bivariate logistic regression. Considering the internal relation among MLD, RLD, %DS and LL/MLD^4^, the four parameters were not combined in one model. Therefore, eight combined predictive models were constructed with two lesion parameters as predictors added to QFR.

| **Table 5\|** Logistic regression model with two lesion parameters added to QFR | | | |
| --- | --- | --- | --- |
| **Predictive model** | | **OR (95% CI)** | ***P* value of lesion index** |
| QFR+ | LAD | 0.373 (0.116 - 1.202) | 0.10 |
|  | MLD | 0.058 (0.012 - 0.286) | **<0.0001** |
| QFR+ | LAD | 0.440 (0.147 - 1.314) | 0.14 |
|  | RLD | 0.430 (0.187 - 0.992) | **0.048** |
| QFR+ | LAD | 0.240 (0.075 - 0.765) | **0.02** |
|  | %DS | 1.094 (1.038 - 1.154) | **0.001** |
| QFR+ | LAD | 0.316 (0.091 - 1.092) | 0.07 |
|  | LL/MLD^4^ | 1.216 (1.070 - 1.382) | **0.003** |
| QFR+ | BL | 0.157 (0.046 - 0.542) | **0.003** |
|  | MLD | 0.039 (0.007 - 0.209) | **<0.0001** |
| QFR+ | BL | 0.207 (0.071 - 0.605) | **0.004** |
|  | RLD | 0.299 (0.125 - 0.718) | **0.007** |
| QFR+ | BL | 0.189 (0.060 - 0.595) | **0.004** |
|  | %DS | 1.096 (1.039 - 1.156) | **0.001** |
| QFR+ | BL | 0.073(0.014-0.367) | **0.002** |
|  | LL/MLD^4^ | 1.283(1.100-1.496) | **0.001** |
| *Note.—QFR, quantitative flow ratio; LAD, lesion in left anterior descending artery; BL, side branch at the lesion site; MLD, minimal lumen diameter; RLD, reference lumen diameter; %DS, percent diameter stenosis; OR, odd ratio; CI, confidence interval.* | | | |

# SI Appendix VI: Performance of combined models on patients with stable angina

The accuracy, sensitivity, specificity, PPV and NPV of the five combined models are presented in Table 6 (the other three were not effective). In contrast to QFR, the AUCs of QFR+LAD+%DS, QFR+BL+MLD, QFR+BL+RLD, QFR+BL+%DS and QFR+BL+LL/MLD^4^ were all significantly improved (0.90 vs. 0.85, P = 0.03; 0.92 vs. 0.85, P = 0.005; 0.90 vs. 0.85, P = 0.03; 0.93 vs. 0.85, P = 0.004, respectively) without compromising other performance (Table 6). Notably, the combined QFR+BL+MLD model outperformed QFR with significant higher accuracy (91.38 vs. 81.90, P = 0.03). In addition, the sensitivity, specificity, PPV and NPV of the combined QFR+BL+MLD model were higher than those of QFR, but they were not statistically significant (both P > 0.05).

| **Table 6\|** Performance of combined models on patients with stable angina | | | | | | |
| --- | --- | --- | --- | --- | --- | --- |
| **Predictive model** | **AUC** | **Accuracy**  **(%)** | **Sensitivity**  **(%)** | **Specificity**  **(%)** | **PPV**  **(%)** | **NPV**  **(%)** |
| QFR | 0.85 | 81.90 | 80.39 | 83.08 | 78.85 | 84.38 |
| QFR+LAD+%DS | 0.90 | 87.07 | 80.39 | 92.31 | 89.13 | 85.71 |
| QFR+BL+MLD | 0.92 | 91.38 | 90.20 | 92.31 | 90.2 | 92.31 |
| QFR+BL+RLD | 0.90 | 83.62 | 88.23 | 80.00 | 77.59 | 89.66 |
| QFR+BL+%DS | 0.90 | 85.34 | 92.16 | 80.00 | 78.33 | 92.86 |
| QFR+BL+LL/MLD^4^ | 0.93 | 89.66 | 86.27 | 92.31 | 89.8 | 89.55 |
| *Note.—QFR, quantitative flow ratio; LAD, lesion in left anterior descending artery; MLD, minimal lumen diameter; RLD, reference diameter; %DS, diameter stenosis; BL, side branch at lesion site; AUC, area under the receiver operating characteristic curve; PPV, positive predictive value; NPV, negative predictive value.* | | | | | | |

As for the patients with unstable angina, QFR correctly classified 32 of the 36 vessels with 16 true positives, 16 true negatives, 1 false negative and 3 false positives. Due to the lack of enough samples, there is little point to construct combined models on patients with unstable angina in the present study. In addition, none of the selected parameters were effective predictors for the combined models. As for the patients with stable angina, the combined QFR+BL+MLD model showed the best performance compared to QFR, which is similar to the analysis without stratification.
